# Characteristics and management of periprosthetic joint infections caused by rapidly growing mycobacteria: a retrospective study and a review of the literature

**DOI:** 10.5194/jbji-9-99-2024

**Published:** 2024-02-29

**Authors:** Pansachee Damronglerd, Eibhlin Higgins, Madiha Fida, Don Bambino Geno Tai, Aaron J. Tande, Matthew P. Abdel, Omar M. Abu Saleh

**Affiliations:** 1 Division of Public Health, Infectious Diseases and Occupational Medicine, Mayo Clinic, 200 First Street SW, Rochester, Minnesota, USA; 2 Division of Infectious Diseases, Faculty of Medicine, Thammasat University, Pathum Thani, Thailand; 3 Division of Infectious Diseases and International Medicine, University of Minnesota, Minneapolis, Minnesota, USA; 4 Department of Orthopedic Surgery, Mayo Clinic, Rochester, Minnesota, USA

## Abstract

**Background**: Periprosthetic joint infection (PJI) following total joint arthroplasty is a serious complication associated with significant morbidity. While Gram-positive cocci are the predominant causative organisms, PJIs caused by rapidly growing mycobacteria (RGM) have been reported, albeit at a lower frequency. This study aimed to investigate the characteristics and management of PJI caused by RGM. **Methods**: A retrospective review was conducted using an institutional PJI database to identify patients diagnosed with PJI due to RGM from January 2010 to December 2021. Clinical data, including demographics, symptoms, comorbidity information, laboratory parameters, surgical procedures, medical treatment and outcomes, were collected and analyzed. **Results**: A total of eight patients were identified with PJI caused by RGM during the study period. The median age was 66 years old, and most cases occurred in patients with total knee arthroplasty ( n=6 ). The isolated RGM species included *Mycobacterium abscessus* (three cases), *M. fortuitum* (three cases), and one case each of *M. immunogenum* and *M. mageritense*. Surgical debridement was performed in all cases, with six patients undergoing two-stage revision and two patients requiring amputation. Combination antimicrobial therapy was administered based on antimicrobial susceptibility testing, and the median duration of treatment was 7.5 months. Adverse events related to therapy occurred in 75 % of cases. No relapses were observed during the median follow-up period of 39.6 months. **Conclusions**: PJI caused by RGM is a rare complication of total joint arthroplasty. Surgical debridement and combination antimicrobial therapy are the mainstays of treatment. Although clinical cure rates are high, amputation may be required in severe cases.

## 1 Introduction

Periprosthetic joint infections (PJIs) are a devastating complication of total joint arthroplasty. The reported incidence of PJI following total hip arthroplasty (THA) and total knee arthroplasty (TKA) is 2.18 % in the United States and 0.97 %–1.03 % in the National Joint Registry, which comprises data from five regions (England, Wales, Northern Ireland, the Isle of Man and New Zealand) (Kurtz et al., 2012). Gram-positive cocci such as *Staphylococcus aureus* and coagulase-negative staphylococci are the most common causative organisms. A prior study from the United States examining microbiological etiology of PJI revealed *Mycobacterium* spp. to be responsible for 0.5 % of infections in their database (Tai et al., 2022), with much higher rates documented in other regions (Jitmuang et al., 2017).

Rapidly growing mycobacteria (RGM) include a subcategory of nontuberculous mycobacteria (NTM) recognized for their ability to develop mature colonies on solid media in less than a week following subculture (Brown-Elliott and Wallace, 2002). These are ubiquitous environmental organisms that can be found in soil, water and aerosols, and they have been linked to a range of clinical conditions, including cutaneous infections, abscesses at injection sites and postsurgical infections (Prevots et al., 2010; Spaulding et al., 2017). Recently, studies have reported an increase in the prevalence of extrapulmonary infections caused by RGM (Cassidy et al., 2009). However, despite this trend, limited data are available on the clinical characteristics and outcomes of PJIs caused by RGM.

Therefore, we conducted a retrospective review of our institutional database to investigate the epidemiology, clinical presentation, management and outcomes of PJIs caused by RGM. In doing so, we aim to contribute to the understanding of the clinical features of PJIs caused by RGM and provide insights into the management of these infections.

## 2 Materials and methods

### 2.1 Study design

We performed a retrospective review of our institutional PJI database to identify patients with PJIs caused by RGM from 1 January 2010 through 31 December 2021.

### 2.2 Case ascertainment and data collection

We used the data from our clinical microbiology lab and the PJI registry database to identify all cases of PJI caused by RGM. We reviewed the patients' medical records after being granted an exempt status by the Mayo Clinic Institutional Review Board. All patients provided consent for the use of their medical records for research purposes. We collected demographic characteristics, clinical symptoms, comorbidity information, laboratory parameters, surgical procedures, medical treatment and outcomes.

### 2.3 Definitions

The diagnosis of PJI was based on the Musculoskeletal Infection Society (MSIS) criteria (Parvizi et al., 2018). The intraoperative diagnostic indicators are positive histology, purulence and a single positive culture. The definitive surgical procedure was defined as either reimplantation as part of two-stage revision or curative amputation.

### 2.4 Laboratory methods

Synovial and sonicate fluid were inoculated into solid (Middlebrook 7H11) and liquid media (BBL^®^ mycobacteria growth indicator tube, MGIT). Periprosthetic tissue specimens were mixed with sterile beef broth and then homogenized using a Stomacher^®^ prior to inoculation into the solid and liquid media. All cultures were incubated at 36±1 °C with a CO_2_ concentration maintained between 5 % and 7 %. Solid cultures were read on a weekly basis over a span of 42 d. Post-incubation, matrix-assisted laser desorption/ionization time-of-flight (MALDI-TOF) analysis was employed for the identification of growth. Susceptibility testing was performed via broth microdilution (BMD) utilizing the commercially available BMD plates by Sensititre™ Myco system (Thermo Fisher Scientific Inc., USA) designed for susceptibility testing for RGM.

## 3 Results

We identified eight patients (50 % female) with PJI due to RGM. The median age was 66 years (range: 49–76 years). Seven cases occurred in patients with total knee arthroplasty. Only one patient (Patient 2) had a history of total hip arthroplasty, four patients had low-grade pain and one patient had history of trauma following implantation. The median time between the implanted prosthesis and the onset of symptoms was 46.5 weeks (range: 2–600 weeks). None of the patients had a known immunodeficiency. Five of the eight patients presented with joint pain and joint swelling. Sinus tract was reported in two patients (patients 2 and 6). Weight loss and diaphoresis were only reported by Patient 5 at initial presentation; Patient 8 presented with fever.

Baseline inflammatory markers were available for seven of eight cases, the median the median erythrocyte sedimentation rate (ESR) at diagnosis was 52 mm h^-1^ (range: 2–104 mm h^-1^) and the serum C-reactive protein (CRP) was 35.2 mg L^-1^ (range: 3–83.9 mg L^-1^). The synovial cell count ranged from 960 to 10 000 cells $µL^{-1}$ , with a predominance of polymorphonuclear cells (range: 60 %–92 %). RGM were isolated from either synovial fluid or deep-tissue specimens. There were three cases of *M. abscessus* PJI, three cases of *M. fortuitum* PJI, and one case each of *M. immunogenum* and *M. mageritense* PJI. Patient characteristics and microbiologic information are summarized in Table 1.

**Table 1:** Demographics of patients in retrospective study.

Case	Age	Underlying	Charlson	Site of	Year of	Symptoms	Organism
	(years),	diseases	comorbidity	prosthesis	implantation		
	sex		index				
1	68, M	HT, CAD, DVT	3	Knee	2006	Pain, discharge from wound	*M. immunogenum*
2	63, F	Obesity	1	Hip	2017	Wound dehiscence, sinus tract, pain	*M. fortuitum*
3	64, M	CKD	2	Knee	2013	Pain, swelling	*M. abscessus*
4	69, M	DJD	2	Knee	2010	Pain, swelling	*M. fortuitum*
5	73, M	TIA, HLP, DJD	4	Knee	2014	Pain, swelling, weight loss, diaphoresis	*M. fortuitum*
6	76, F	PAD, SIADH	3	Knee	2017	Swelling, sinus tract with discharge	*M. abscessus*
7	65, F	Obesity	2	Knee	2012	Pain, swelling	*M. abscessus*
8	49, F	Obesity	1	Knee	2013	Pain, swelling, fever	*M. mageritense*

The abbreviations used in the table are as follows: CKD – chronic kidney disease; DJD – degenerative joint disease; DVT - deep vein thrombosis; HLP – hyperlipidemia; HT – hypertension; PAD – peripheral arterial disease; SIADH – syndrome of inappropriate antidiuretic hormone secretion; TIA – transient ischemic attack.

**Table 2:** Surgical and antimicrobial treatments of patients.

Case	First surgical procedure	Definitive surgical procedure	Antibiotic spacer	Main antimicrobial treatments	Total antimicrobial duration (months)	Outcome
1	Arthroplasty resection with placement static spacer	Amputation	Gentamicin Vancomycin	Amikacin Azithromycin Linezolid Tigecycline	6.3	Remission of infection
2	Revision THA with static spacer change	Reimplantation	Amikacin Vancomycin Amphotericin	Doxycycline Imipenem Minocycline Linezolid Moxifloxacin	11.2	Remission of infection
3	One-stage resection	Reimplantation	Amikacin Gentamicin Vancomycin	Amikacin Cefoxitin Linezolid Tigecycline	14.6	Remission of infection
4	One-stage resection	Reimplantation	No	TMP/SMX Doxycycline Clarithromycin Moxifloxacin	6.9	Remission of infection
5	One-stage revision	Reimplantation	Tobramycin Vancomycin	Ciprofloxacin Imipenem Minocycline	17.1	Remission of infection
6	Revision total knee arthroplasty	Amputation	No	Amikacin Cefoxitin Clofazimine Imipenem Tigecycline	9.4	Remission of infection
7	Arthroplasty revision	Reimplantation	No	Amikacin, Clofazimine Imipenem Tigecycline	10.8	Remission of infection
8	Resection arthroplasty	Reimplantation	No	Clofazimine Imipenem Moxifloxacin Tigecycline	7.5	Remission of infection

The abbreviations used in the table are as follows: THA – total hip arthroplasty; TMP/SMX – trimethoprim–sulfamethoxazole.

### 3.1 Surgical treatment

Surgical debridement was performed in all eight patients. Six of eight patients underwent reimplantation (two-stage revision). Amputation was performed in two (25 %) patients (Table 2). Patient 1 had a history of uncontrolled infection after an exchange spacer, and Patient 6 had a TKA infection with exposed hardware and cultures growing multidrug-resistant *M. abscessus*. In four (50 %) cases, antibiotic spacers containing vancomycin ( n=4 ), amikacin ( n=2 ), gentamicin ( n=2 ) and tobramycin ( n=1 ) were inserted. The median time between the first and the definitive procedure (reimplantation or amputation) was 7.25 months (range: 1.5–25.6 months).

### 3.2 Antimicrobial treatment

**Figure 1:**
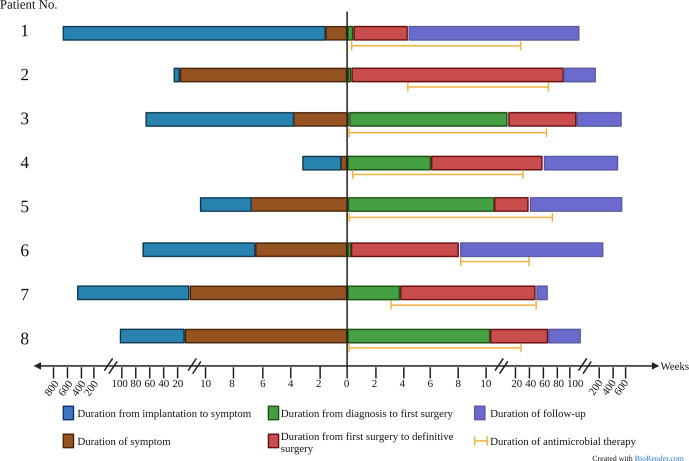
Timeline of eight patients in the retrospective study.

All patients were treated with a combination of parenteral and oral antimicrobial chemotherapy based on antimicrobial susceptibilities. Antimicrobial therapy was started just before or at the time of the first surgical intervention in six out of eight patients. In two patients, therapy was initiated later – due to delayed microbial diagnosis in one case and until the time of amputation in the other case, which was first surgical intervention directed at the mycobacterial PJI. Clofazimine was part of the regimen in three patients (patients 6, 7 and 8) because of extensive antimicrobial resistance. The median duration of antimicrobial therapy was 10.5 months (range: 6.3–17.1 months). The duration was determined based on several factors, including the timing of definitive surgical treatment, the nature of the surgical intervention and the specific RGM species causing the infection. Five out of six patients who underwent a reimplantation discontinued antimicrobial treatment both before and after the definitive operation. The timeline of various events during management is shown in Fig. 1, and antimicrobial susceptibility data are summarized in Table 3.

**Table 3:** Selected antimicrobial susceptibilities of rapidly growing mycobacteria causing prosthetic joint infections.

Case	Organism	Amikacin	Cefoxitin	Ciprofloxacin	Clarithromycin	Clofazimine	Doxycycline	Imipenem	Linezolid	Minocycline	Moxifloxacin	Tigecycline	Tobramycin	TMP/SMX
1	*M. immunogenum*	16, S	16, S	>4 , R	0.5, S		>16 , R	8, I	16, I	>8 , R	>8 , R	0.25, S	16, R	8/152, R
2	*M. fortuitum*	2, S	64, I	≤0.12 , S	8, R		0.25, S	≤2 , S	2, S	≤1 , S	≤0.25 , S	0.12, S	>16 , R	≤0.25/4.75 , S
3	*M. abscessus*	16, S	32, I	>4 , R	8, R		>16 , R	8, I	16, I		>8 , R	0.12, S	16, R	>8/152 , R
4	*M. fortuitum*	S	I	S	I		R	S	R		S	S	S	S
5	*M. fortuitum*	4, S	32, I	≤0.12 , S	>16 , R		0.25, S	≤2 , S	2, S		≤0.25 , S	0.06, S	>16 , R	1/19 , S
6	*M. abscessus*	8, S	32, I	>4 , R	>16 , R	0.16	>16 , R	8, I	32, R	>8 , R	>8 , R	0.25, S	8, R	>8/152 , R
7	*M. abscessus*	16, S	64, I	>4 , R	>16 , R	0.5	>8 , R	16, I	>32 , R		>4 , R	1, S	8, R	>4/76 , R
8	*M. mageritense*	4, S	64, I	0.25, S	>16 , R		8, R	8, I	8, I		≤0.25 , S	1, S		>8/152 , R

The results are presented as follows: $µg\;{\mathrm{mL}}^{-1}$ , S/I/R, where S represents susceptible, I represents intermediate and R represents resistant.TMP/SMX denotes trimethoprim–sulfamethoxazole.

### 3.3 Adverse events

Therapy-related adverse events occurred in 75 % of cases; this led to a change in the treatment regimen. The most common event was nausea and vomiting. Neurotoxicity was also reported. There was one case of amikacin-related acute kidney injury and one case of ototoxicity. Amikacin-related hearing loss occurred after 4 months of parenteral therapy. There were two cases of fluoroquinolone-related adverse events including QTc prolongation in one patient and joint pain in another patient. Minocycline caused cutaneous hyperpigmentation in one case after 5 months of therapy. One patient developed a *Clostridioides difficile* infection 1 month after stopping therapy.

### 3.4 Outcomes

None of the patients developed clinical or microbiological relapse after a combination of surgical and antimicrobial intervention. The median of duration of follow-up was 39.6 months (range: 10.6–78 months). As mentioned above, surgical intervention included reimplantation in six patients and amputation in another two patients.

**Table 4:** Articles included in the review of the literature.

Author and year of publication	Joint involvement (no. of cases)	Pathogens	Surgical intervention	Outcome
Badelon et al. (1979)	Hip (3)	*M. fortuitum* (3)	Resection arthroplasty (1), debridement with retained prosthesis (2)	Persistent infection (2), not reported (1)
Eid et al. (2007)	Knee (7), elbow (1), hip (1)	*M. chelonae* (3), *M. fortuitum* (3), *M. smegmatis* (1), *M. abscessus* (1)	Resection arthroplasty followed by reimplantation (2), resection arthroplasty (5), DAIR (2)	Cured (4), receiving long-term suppressive therapy (3), palliative care (1)
Henry et al. (2016)	Knee (1), hip (1)	*M. smegmatis* (1), *M. abscessus* (1)	Resection arthroplasty followed by reimplantation (2)	Cured (2)
Jitmuang et al. (2017)	Knee (10), hip (1)	*M. fortuitum* (9), *M. abscessus* (1), *M. peregrinum* (1)	Resection arthroplasty followed by reimplantation (9), resection arthroplasty (1), debridement with retained prosthesis (1)	Favorable at 6 months (6), unfavorable at 6 months (3), not reported (2)
Kim et al. (2017)	Knee (2)	*M. abscessus* (2)	Resection arthroplasty followed by reimplantation (2)	Cured (2)
Goldstein et al. (2019)	Hip (2)	*M. abscessus* (1), *M. fortuitum* (1)	Resection arthroplasty followed by delayed reimplantation (1), amputation (1)	Cured (2)

DAIR represents debridement, antibiotics and implant retention

### 3.5 Review of the literature

A comprehensive review of the available literature revealed six published case series that collectively reported 29 cases with PJIs caused by RGM (Table 4). Among these cases, the sites of infection included 20 knees, 8 hips and 1 elbow, with 1 patient having 2 joints infected. The specific RGM species involved were as follows: *M. fortuitum* (16 cases), *M. abscessus* (6 cases), *M. chelonae* (3 cases), *M. smegmatis* (2 cases) and *M. peregrinum* (1 case). Resection arthroplasty was the most common primary surgical intervention in 22 of 29 PJIs, followed by delayed reimplantation in 16 of 29 cases. DAIR (debridement, antibiotics and implant retention) was performed in 5 of 29 episodes and amputation was pursued in 2 episodes. The duration of antimicrobial treatment was varied in each case, ranging from 0.5 to 30.3 months, with a median of 9 months. In two cases, the duration of treatment was not reported. Favorable outcomes (16 cases) were reported in the majority of patients who underwent complete two-stage revision.

## 4 Discussion

This study evaluated patients diagnosed with PJI caused by RGM. Over an 11-year period, we identified eight cases of PJI caused by RGM, including the first and the second reported case of PJI caused by *M. immunogenum* and *M. mageritense*, respectively. All patients had undergone total arthroplasty to treat degenerative joint disease (DJD). None of the patients had any known immunodeficiencies, which are typically associated with an increased risk of RGM infection. The interval from prosthesis implantation to symptom onset in this study varied widely, similar to findings reported in Colorado, USA, (8.5 months) (Goldstein et al., 2019) but shorter than a previous Mayo Clinic report (78 months) (Eid et al., 2007). In their cohort, Jitmuang et al. (2017) found that early-onset PJI (within 60 d of primary arthroplasty) was significantly associated with RGM infection.

A consistent observation in the previously published literature would suggest that PJI with rapidly growing mycobacteria is more commonly reported with total knee arthroplasty compared with hip arthroplasty, as was also observed in our study; this might be related to their higher vulnerability to exogenous trauma and inoculation secondary to proximity to the overlying soft tissue envelope.

Given the chronic nature of RGM PJI and the difficult-to-treat nature of the isolate pathogens, it is not surprising that the most pursued surgical intervention was an initial resection arthroplasty, followed by delayed reimplantation in selected patients.

In our study, the duration of antimicrobial therapy for patients with PJI caused by RGM ranged from 6.3 to 17.1 months. The selection of antimicrobial therapy to treat PJI caused by RGM should be based on the results of antimicrobial susceptibility testing. Given the rarity of RGM PJI, the evidence guiding the therapy is limited and has largely been extrapolated from NTM pulmonary infection (Kurz et al., 2020). Treatment requires a multimodal approach, including surgical debridement, arthroplasty resection and prolonged combination antimicrobial therapy.

Antimicrobial resistance and treatment-related toxicity pose significant challenges in the management of NTM infections. Treatment-related adverse events were reported in 75 % of the patients included in our cohort. Due to the multidrug-resistant nature of some of the RGM, we noted an increase in the utilization of agents like clofazimine.

Clofazimine was utilized as part of the combination regimen in two patients with macrolide-resistant *M. abscessus* PJIs and in one patient with macrolide-resistant *M. mageritense* PJI. In vitro studies have shown that clofazimine exhibits synergistic effects with amikacin in the treatment of nontuberculous mycobacterial disease (Van Ingen et al., 2012). Despite its efficacy, clofazimine is known to cause adverse effects, such as skin discoloration and gastrointestinal intolerance (Cariello et al., 2015), which were also observed in two patients in this series.

*Mycobacterium immunogenum* is a novel species closely related to the *M. abscessus* group. This organism was first identified in 2001 as a contaminant in metalworking fluids, and it has been associated with pseudo-outbreaks (Wilson et al., 2001; Gordon et al., 2006). Since then, numerous case reports of infections caused by *M. immunogenum* have been described, with the most common manifestation being cutaneous disease. This can take the form of chronic ulcers, eruptive erythematous pustules or nodules, which can develop following exposure to various sources, such as mesotherapy, hot tubs, surgical sites and tattoos (Loots et al., 2005; Garcia-Zamora et al., 2017; McNeil et al., 2019). In our patient's case, no clear exposure risk factor was identified. Resistance to antibiotics is a concern with *M. immunogenum*, with the isolate in this study showing resistance to ciprofloxacin, doxycycline, minocycline, tobramycin, moxifloxacin and trimethoprim–sulfamethoxazole. Similarly, Wilson et al. (2001) found cefoxitin and tobramycin resistance in their study. Our patient was treated for 6 months with a combination of amikacin, tigecycline, azithromycin and linezolid for intramedullary osteomyelitis following amputation. Although there is a lack of consensus regarding the optimal treatment regimen for *M. immunogenum* infections, this combination therapy with amikacin, azithromycin, linezolid and tigecycline was successful in treating our patient's infection.

*Mycobacterium mageritense* is a type of nonpigmented RGM that shares similarities with the *M. fortuitum* complex. It was first discovered in Spain in 1987 and identified as a unique species by Domenech et al. (1997) in 1997. Although human infections caused by *M. mageritense* are rare, several case reports have described skin and soft tissue infections, particularly surgical site infections, as well as device-related infections, including catheter-related bloodstream infections (Koyama et al., 2021), prosthetic valve endocarditis (McMullen et al., 2015) and abdominal mesh infections (Ando et al., 2021). In 2020, the first case of *M. mageritense* PJI was reported, where the patient had undergone revision surgery of knee due to hardware loosening. Subsequently, the patient developed wound dehiscence and fluid collection. The isolated strain of *M. mageritense* in this case was susceptible to amikacin, ciprofloxacin, imipenem, linezolid, minocycline, moxifloxacin and trimethoprim–sulfamethoxazole; showed intermediate susceptibility to cefoxitin and doxycycline; and displayed resistance to clarithromycin (Caravedo Martinez and Blanton, 2020). In contrast, our isolated strain of *M. mageritense* was susceptible to amikacin, ciprofloxacin, moxifloxacin and tigecycline. We successfully treated our patient with a combination of imipenem, moxifloxacin, tigecycline and clofazimine for a duration of approximately 7 months, along with a two-stage revision.

## 5 Conclusions

This study highlights the clinical features, treatment approaches and outcomes of RGM PJI – an exceedingly rare complication of total joint arthroplasty. Therapeutic strategy in this cases series utilized surgical intervention in tandem with combination antimicrobial therapy. The combination pharmacotherapy required has significant associated toxicity. Thus, close clinical monitoring along with collaboration with pharmacy colleagues is essential to detect and address adverse drug events. Whilst clinical cure rates were high in this series, amputation was required for source control in 25 % of cases, highlighting the significant morbidity associated with this infection.

## Data Availability

The datasets generated during and/or analyzed during the current study are available from the corresponding author upon reasonable request.

## References

[bib1.bib1] Ando J, Miyata R, Harada M, Takeuchi M, Kasahara K, Yoshimoto Y, Koyama F, Kuwahara M (2021). A Ventral Hernia-repair-related Mycobacterium mageritense Mesh Infection Treated with NPWT without Mesh Removal. Plast Reconstr Surg Glob Open.

[bib1.bib2] Badelon O, David H, Meyer L, Radault A, Zucman J (1979). [Mycobacterium fortuitum infection after total hip prosthesis. A report of 3 cases (author's transl.)]. Rev Chir Orthop Reparatrice Appar Mot.

[bib1.bib3] Brown-Elliott BA, Wallace Jr RJ (2002). Clinical and taxonomic status of pathogenic nonpigmented or late-pigmenting rapidly growing mycobacteria. Clin Microbiol Rev.

[bib1.bib4] Caravedo Martinez MA, Blanton LS (2020). Mycobacterium mageritense Prosthetic Joint Infection. Case Rep Infect Dis.

[bib1.bib5] Cariello PF, Kwak EJ, Abdel-Massih RC, Silveira FP (2015). Safety and tolerability of clofazimine as salvage therapy for atypical mycobacterial infection in solid organ transplant recipients. Transpl Infect Dis.

[bib1.bib6] Cassidy PM, Hedberg K, Saulson A, McNelly E, Winthrop KL (2009). Nontuberculous mycobacterial disease prevalence and risk factors: a changing epidemiology. Clin Infect Dis.

[bib1.bib7] Domenech P, Jimenez MS, Menendez MC, Bull TJ, Samper S, Manrique A, Garcia MJ (1997). Mycobacterium mageritense sp. nov. Int J Syst Bacteriol.

[bib1.bib8] Eid AJ, Berbari EF, Sia IG, Wengenack NL, Osmon DR, Razonable RR (2007). Prosthetic joint infection due to rapidly growing mycobacteria: report of 8 cases and review of the literature. Clin Infect Dis.

[bib1.bib9] Garcia-Zamora E, Sanz-Robles H, Elosua-Gonzalez M, Rodriguez-Vasquez X, Lopez-Estebaranz JL (2017). Cutaneous infection due to Mycobacterium immunogenum: an European case report and review of the literature. Dermatol Online J.

[bib1.bib10] Goldstein N, St Clair JB, Kasperbauer SH, Daley CL, Lindeque B (2019). Nontuberculous Mycobacterial Musculoskeletal Infection Cases from a Tertiary Referral Center, Colorado, USA. Emerg Infect Dis.

[bib1.bib11] Gordon T, Nadziejko C, Galdanes K, Lewis D, Donnelly K (2006). Mycobacterium immunogenum causes hypersensitivity pneumonitis-like pathology in mice. Inhal Toxicol.

[bib1.bib12] Henry MW, Miller AO, Kahn B, Windsor RE, Brause BD (2016). Prosthetic joint infections secondary to rapidly growing mycobacteria: Two case reports and a review of the literature. Infect Dis (Lond).

[bib1.bib13] Jitmuang A, Yuenyongviwat V, Charoencholvanich K, Chayakulkeeree M (2017). Rapidly-growing mycobacterial infection: a recognized cause of early-onset prosthetic joint infection. BMC Infect Dis.

[bib1.bib14] Kim M, Ha CW, Jang JW, Park YB (2017). Rapidly growing non-tuberculous mycobacteria infection of prosthetic knee joints: A report of two cases. Knee.

[bib1.bib15] Koyama T, Funakoshi Y, Imamura Y, Nishimura S, Fujishima Y, Toyoda M, Kiyota N, Tanino H, Minami H (2021). Device-related Mycobacterium mageritense Infection in a Patient Treated with Nivolumab for Metastatic Breast Cancer. Intern Med.

[bib1.bib16] Kurtz SM, Lau E, Watson H, Schmier JK, Parvizi J (2012). Economic burden of periprosthetic joint infection in the United States. J Arthroplasty.

[bib1.bib17] Kurz SG, Zha BS, Herman DD, Holt MR, Daley CL, Ruminjo JK, Thomson CC (2020). Summary for Clinicians: 2020 Clinical Practice Guideline Summary for the Treatment of Nontuberculous Mycobacterial Pulmonary Disease. Ann Am Thorac Soc.

[bib1.bib18] Loots MA, de Jong MD, van Soolingen D, Wetsteyn JC, Faber WR (2005). Chronic leg ulcer caused by Mycobacterium immunogenum. J Travel Med.

[bib1.bib19] McMullen AR, Mattar C, Kirmani N, Burnham CA (2015). Brown-Pigmented Mycobacterium mageritense as a Cause of Prosthetic Valve Endocarditis and Bloodstream Infection. J Clin Microbiol.

[bib1.bib20] McNeil EP, Goldfarb N, Hannon GR, Miller DD, Farah RS (2019). Mycobacterium immunogenum folliculitis on the lower extremities of a healthy young adult. Clin Exp Dermatol.

[bib1.bib21] Parvizi J, Tan TL, Goswami K, Higuera C, Della Valle C, Chen AF, Shohat N (2018). The 2018 Definition of Periprosthetic Hip and Knee Infection: An Evidence-Based and Validated Criteria. J Arthroplasty.

[bib1.bib22] Prevots DR, Shaw PA, Strickland D, Jackson LA, Raebel MA, Blosky MA, Montes de Oca R, Shea YR, Seitz AE, Holland SM, Olivier KN (2010). Nontuberculous mycobacterial lung disease prevalence at four integrated health care delivery systems. Am J Respir Crit Care Med.

[bib1.bib23] Spaulding AB, Lai YL, Zelazny AM, Olivier KN, Kadri SS, Prevots DR, Adjemian J (2017). Geographic Distribution of Nontuberculous Mycobacterial Species Identified among Clinical Isolates in the United States, 2009–2013. Ann Am Thorac Soc.

[bib1.bib24] Tai DBG, Patel R, Abdel MP, Berbari EF, Tande AJ (2022). Microbiology of hip and knee periprosthetic joint infections: a database study. Clin Microbiol Infect.

[bib1.bib25] van Ingen J, Totten SE, Helstrom NK, Heifets LB, Boeree MJ, Daley CL (2012). In vitro synergy between clofazimine and amikacin in treatment of nontuberculous mycobacterial disease. Antimicrob Agents Chemother.

[bib1.bib26] Wilson RW, Steingrube VA, Bottger EC, Springer B, Brown-Elliott BA, Vincent V, Jost KC, Zhang Y, Garcia MJ, Chiu SH, Onyi GO, Rossmoore H, Nash DR, Wallace RJ (2001). Mycobacterium immunogenum sp. nov., a novel species related to Mycobacterium abscessus and associated with clinical disease, pseudo-outbreaks and contaminated metalworking fluids: an international cooperative study on mycobacterial taxonomy. Int J Syst Evol Microbiol.

